# Association of SNPs in the FK-506 binding protein (*FKBP5*) gene among Han Chinese women with polycystic ovary syndrome

**DOI:** 10.1186/s12920-022-01301-0

**Published:** 2022-07-04

**Authors:** Xinyue Ma, Zhao Wang, Changming Zhang, Yuehong Bian, Xin Zhang, Xin Liu, Yongzhi Cao, Yueran Zhao

**Affiliations:** 1 Center for Reproductive Medicine, Cheeloo College of Medicine, Shandong University, Jinan, 250012 Shandong China; 2 National Research Center for Assisted Reproductive Technology and Reproductive Genetics, Shandong University, Jinan, 250012 Shandong China; 3 Key Laboratory of Reproductive Endocrinology of Ministry of Education, Shandong University, Jinan, 250012 Shandong China; 4 Shandong Provincial Clinical Medicine Research Center for Reproductive Health, Shandong University, Jinan, 250012 Shandong China; 5 Central Laboratory, Shandong Provincial Hospital, Cheeloo College of Medicine, Shandong University, Jinan, 250021 Shandong China

**Keywords:** PCOS, Hyperandrogenism, *FKBP5*, SNP, Association

## Abstract

**Background:**

Polycystic ovary syndrome (PCOS) is a common endocrine disorder in premenopausal women, whose etiology remains uncertain, although it is known to be highly heterogeneous and genetically complex. PCOS often presents with hyperandrogenism symptoms. The present study aimed to determine whether polymorphisms in the FK-506 binding protein 5 (*FKBP5*) gene (androgen target gene) are associated with an association for PCOS and hyperandrogenism.

**Methods:**

This is a case–control study, and association analyses were conducted. A total of 13 single-nucleotide polymorphisms (SNPs) in the *FKBP5* gene were evaluated in 775 PCOS patients who were diagnosed based on the Rotterdam Standard and 783 healthy Chinese Han women. Associations between *FKBP5* SNPs and hormone levels were investigated. These 13 SNPs were genotyped using the Sequenom MassARRAY system, and an association analysis between the phenotype and alleles and genotypes were conducted.

**Results:**

The genotype frequencies for the rs1360780 and rs3800373 SNPs differed significantly between the PCOS cases and healthy controls (p = 0.025, OR is 1.63 (1.05–2.53) and p = 0.029, OR is 1.59 (1.03–2.45) respectively under co-dominant model). Moreover, the genotype frequencies and genetic model analysis for the SNPs rs1360780, rs9470080, rs9296158, rs1043805 and rs7757037 differed significantly between the hyperandrogenism and non-hyperandrogenism groups of PCOS patients. The TT genotype of rs1360780, the TT genotype of rs9470080, the TT genotype of rs1043805 or the GG genotype of rs7705037 (ORs are 2.13 (1.03–4.39), 1.81 (1.03–3.17), 2.94 (1.32–6.53) and 1.72 (1.04–2.84) respectively) were correlated with androgen level of PCOS patients.

**Conclusion:**

Our study showed that *FKBP5* gene polymorphisms are associated with PCOS generally (rs1360780 and rs3800373) and with the hyperandrogenism subtype specifically (rs1360780, rs9470080, rs9296158, rs1043805 and rs7757037).

**Supplementary Information:**

The online version contains supplementary material available at 10.1186/s12920-022-01301-0.

## Background

Polycystic ovary syndrome (PCOS) is an endocrine disorder which afflicts females of reproductive age [1]. While the etiology of PCOS remains unclear, PCOS patients differ from healthy women in many aspects, often presenting with chronic anovulation (ovulation dysfunction or loss), insulin resistance, polycystic ovarian morphology (PCOM) under B-ultrasound (multiple cysts are formed in the ovarian follicles), and hyperandrogenism (high levels of androgens in females) [2]. Notably, interplay between hyperandrogenism and insulin resistance has been reported to influence the occurrence and development of PCOS [3]. Disrupted secretion of the pulsatile gonadotropin-releasing hormone (GnRH) from the hypothalamus has also been associated with PCOS, as have imbalances in regulation of both the hypothalamic–pituitary–adrenal (HPA) axis and the hypothalamic–pituitary–ovary (HPO) axis [3, 4]. High androgen exposure during the embryonic period, endocrine disorders, and immune dysregulation have, among other factors, also been linked to PCOS [5, 6].

PCOS is considered to be a chronic inflammatory disease [1], and multiple inflammation-related genes (such as interleukin-1 beta (*IL1B*), prostaglandin-endoperoxide synthase 2 (*PTGS2*)), as well as granule cells(GCs) in the inflammatory environment of PCOS patients, have been linked to hyperandrogenism [7–9]. Hyperandrogenism is understood to damage the health of PCOS patients, and hyperandrogenism is now an important criterion for PCOS diagnosis. The most common clinical manifestation of hyperandrogenism in women is hirsutism and excessive terminal hair growth in androgen-dependent areas of the body. Other clinical manifestations of hyperandrogenism include acne vulgaris, weight gain, menstrual irregularities, and acanthosis nigricans [10]. The incidence rate of hyperandrogenism is as high as 60–80% [11, 12]. Androgens can be biosynthesized from cholesterol by the theca cells in the ovary [13]; these hormones function in regulating metabolic homeostasis and reproductive health in both men and women. In PCOS, excess androgens lead to follicular dysplasia and anovulatory infertility, with studies suggesting that theca cell androgen receptors likely mediate many of these negative effects [14]. Moreover, disrupted androgen levels have been linked to other clinical manifestations common to PCOS patients, including obesity, type 2 diabetes, hypertension and atherosclerosis, cardiac hypertrophy, and coronary heart disease, as well as kidney diseases [11].

PCOS has a strong genetic component [15]. Some SNPs of PCOS-related genes are partially associated with the morbidity of PCOS [16–18]. For example, the GG genotype of *LHCGR* (rs2293275) in PCOS showed strong correlations with body-mass index (BMI), waist to hip ratio, insulin resistance, luteinizing hormone, and LH/FSH ratio [19]. Moreover, transmission disequilibrium testing revealed a strong genetic association between the D19S884 allelic marker located near the *INSR* gene and the development of PCOS. Additionally, a T/C polymorphism was also reported among the Chinese population in the *CYP17A* gene: affected females having the CC genotype had increased testosterone levels compared to individuals harboring the TC and TT genotypes. Additional genes with PCOS-associations include the androgen receptor (*AR*) gene, the sex hormone-binding globulin (*SHBG*) gene, and the insulin-like factor 3 (*INSL3*) gene [20].

*FKBP5* encodes the FK-506 binding protein 5 (a co-chaperone of a heat shock protein (Hsp90)) [21], which is known to be directly regulated by androgens and has been implicated in metabolism-related disorders. Specifically, there is a distal enhancer located 65 kb downstream of the transcription start site in the fifth intron of the *FKBP5* gene, and a study showed that *FKBP5* expression is regulated by an interaction between the AR and this enhancer; specifically, AR selectively recruits cAMP response element-binding protein to this enhancer [22]. Moreover, it is notable that previous studies have shown that *FKBP5* is highly expressed in muscle and adipose tissue, and human *FKBP5* is associated with type 2 diabetes(T2D) and with markers for both insulin resistance and obesity [23, 24]. Given that PCOS patients show symptoms like endocrine disorders, metabolic disorders, and obesity, we were interested in exploring potential relationships between *FKBP5* and androgens in the context of PCOS etiology. The objective of the present case–control study was to investigate potential associations between single nucleotide polymorphisms of *FKBP5* and PCOS pathogenesis and symptoms.

## Methods

### Ethics statement

All patients in this study gave their informed written consent; the protocol for this study was reviewed and approved by the Institutional Review Board of Reproductive Medicine of Shandong University ([2020] Ethical Review #44).

### Study subjects

A total of 1558 women from the Reproductive Hospital affiliated with Shandong University were included in the present case–control study. Among them, 775 women had PCOS, while the other 783 were healthy control subjects, whose blood and other samples have been kept in the sample bank of the Affiliated Reproductive Hospital of Shandong University for research. The patients with PCOS and the healthy control subjects were diagnosed based on the presence of two out of three criteria of proposed by the Rotterdam European Society for Human Reproduction and Embryology (ESHRE)/American Society for Reproductive Medicine (ASRM)-sponsored PCOS Consensus Workshop Group; patients with PCOS were included if they met at least two of the following three criteria: (1) chronic oligoovulation and/or anovulation; (2) clinical or biochemical hyperandrogenism; and (3) polycystic ovaries on ultrasound, ≥ 12 ovary follicles measuring 2–9 mm in diameter in one or both ovaries, or ovarian volume > 10 mL [25]. Other etiologies (congenital adrenal hyperplasias, androgen-secreting tumors, and Cushing's syndrome) were excluded. According to the level of androgen, there were 588 cases are with hyperandrogenism subtype; and 187 cases were of the non-hyperandrogenism subtype. Clinical or biochemical hyperandrogenism was defined on the basis of hirsutism (modified Ferriman-Gallwey score ≥ 6) or elevated circulating total testosterone ≥ 60 ng/dl [26, 27].

Controls were gathered primarily from healthy women who presented with regular menstrual cycles, excluding hyperandrogenism and PCOS. Clinical parameters of the patients with PCOS and the healthy control subjects are shown in Table 1.

**Table 1:** Clinical and endocrine characteristics of the healthy control subjects and patients with polycystic ovary syndrome (PCOS)

	PCOS (n = 775)	Control (n = 783)	P
Ages (years)	30.26 ± 0.19	30.25 ± 0.16	Ns
BMI (kg/m^2^)	24.40 ± 0.17	22.72 ± 0.13	< 0.0001
WC	85.05 ± 0.50	78.18 ± 0.30	< 0.0001
WHR	0.88 ± 0.00	0.84 ± 0.00	< 0.0001
FSH (IU/L)	6.16 ± 0.07	8.78 ± 0.37	< 0.0001
LH (IU/L)	12.86 ± 0.31	4.94 ± 0.19	< 0.0001
LH/FSH	2.13 ± 0.05	0.61 ± 0.02	< 0.0001
T (ng/dL)	72.65 ± 1.20	44.25 ± 0.87	< 0.0001

Independent sample t-test; data are expressed as means ± SD

*PCOS* polycystic ovary syndrome, *BMI* body mass index, *WC* waist circumference, *WHR* waist-hip ratio, *FSH* follicle-stimulating hormone, *LH* luteinizing hormone, *T* testosterone

### Measurement of clinical data

The patients with PCOS and the healthy control subjects were diagnosed and examined at the Center for Reproductive Medicine, Shandong Provincial Hospital, Shandong University, China. Weight and height were measured using standard protocols with calibrated instruments. Body mass index (BMI) was calculated as weight (kg) divided by height squared (m^2^). Peripheral blood samples were collected on days 2–5 of a spontaneous cycle or after withdrawal of bleeding from the subjects in a fasting state. Measurement with a chemiluminescent analyzer (Beckman Access Health Company, Chaska, Minnesota, USA) was done for the following hormones: follicle-stimulating hormone (FSH), luteinizing hormone (LH), and testosterone (T). Blood samples, which were collected with EDTA as an anticoagulant and stored at − 80 °C, were prepared for genomic DNA extraction.

### Clinical data of the PCOS Group and the control group

We analyzed subjects from the two groups (PCOS group and control group); the clinical characteristics of groups are shown in Table 1. The mean age did not different between the two groups. BMI and WHR were significantly higher in the PCOS group than in the control group (p < 0.001). The levels of LH, LH/FSH, and T in the PCOS group were higher in the PCOS group than in the controls. The FSH level was significantly lower in the PCOS group compared to the controls.

### SNP genotyping

To investigate potential associations between single nucleotide polymorphisms of *FKBP5* and PCOS pathogenesis and symptoms, for the total 54,836 variants in the *FKBP5* gene, we chose 13 SNPs. Selected criteria: functional region SNP were selected, including promoter, exon and 3′ UTR of gene, by SNP Function Prediction (https://snpinfo.niehs.nih.gov/) [28] to evaluate potential functional mechanism, this step includes 3 functional region SNPs: rs3800373 and rs1043805 belonging to the 3′ UTR region, and rs2817035 belonging to the promoter region. Furthermore, we searched literature to filter known important risk SNP of genes, for our sample validation, this step incorporates the remaining 10 SNPs. MAF of above all SNP were verified through 1000 Genomes CHB (https://www.internationalgenome.org/), setting up cutoff MAF > 0.05. Among them, the SNPs rs2817035 and rs4713902 of the *FKBP5* gene were reported as linked to risk for coronary artery disease [24]. A study of interindividual response differences to inhaled corticosteroids in patients with chronic obstructive pulmonary disease showed that rs4713916 is associated with sensitivity/resistance to corticosteroids [29]. Considering that rs1360780, rs3800373, rs9296158, and rs9470080 are commonly detected SNPs of the *FKBP5* gene [30], we focused on these SNPs in this study. In total, we selected 13 SNPs in *FKBP5* (Table 2).

**Table 2:** Genomic information of selected SNPs of FKBP5 genotyped in this study

SNPs	Allele		Frequency (1000Genomes CHB)	Position	Region
	Major	Minor			
rs1360780	C	T	A = 0.26/1341	6:35,728,586	Intron
rs3800373	A	C	C = 0.25/1328	6:35,574,699	3’ UTR
rs9296158	G	A	A = 0.32/1340	6:35,599,305	Intron
rs9470080	C	T	T = 0.33/1327	6:35,678,658	Intron
rs2817035	G	A	A = 0.23/1341	6:35,728,586	Promoter
rs3798346	A	G	G = 0.05/1318	6:35,594,863	Intron
rs4713902	T	C	C = 0.26/1340	6:35,646,249	Intron
rs4713916	G	A	A = 0.22/1341	6:35,702,206	Intron
rs755658	C	T	T = 0.08/1341	6:35,581,893	Intron
rs7757037	A	G	A = 0.39/1340	6:35,580,459	Intron
rs9394309	A	G	G = 0.21/1340	6:35,654,004	Intron
rs1043805	A	T	T = 0.18/1340	6:35,573,655	3′ UTR
rs1475774	G	A	A = 0.07/1325	6:35,651,777	Intron

The frequency information is from the samples of this study and the position information of SNPs is on the basis of GRCh38.p12

The genomic DNA of every subject was extracted from peripheral blood using DNA Blood Mini Kits (QIAGEN, 51,106, Germany). Thirteen SNPs in the *FKBP5* gene were genotyped using the MassARRAY RS1000 platform (Sequenom, San Diego, CA, USA) according to the standard protocol. The genotyping primers were designed using MassARRAY Assay Design 3.1 Software (Sequenom) and were synthesized by Biomiao Biological Technology Company (Beijing, China). Sequencing was performed on a MassARRAY Compact System (Sequenom), and the genotype data were analyzed using MassARRAY TYPER Analyzer Software version 4.0 (Sequenom).

### Statistical analysis

Tests of Hardy–Weinberg equilibrium and linkage disequilibrium analysis were performed using HaploView software. The analysis of the association between clinical characteristics and genotypes was performed with independent sample t tests. The allele frequencies and genotype frequencies of the 13 SNPs were tested with chi-square tests, and were adjusted using logistic regression analysis [31]. The genotype frequency of the 13 SNPs were analyzed in terms of five genetic models using SNPStats software (http://bioinfo.iconcologia.net/snpstats/start.htm): the log-additive model (+/+ vs. −/−), the dominant model (+/+ plus +/− vs. −/−), the recessive model (+/+ vs. +/− plus −/−), the co-dominant model (+/+ vs. +/− vs. −/−), and the overdominant model (+/+ plus −/− vs. +/−). Considering that the outcome variables are dichotomous (PCOS and control, hyperandrogenism and non-hyperandrogenism), the log-additive model was performed. The genetic model was assessed using the Akaike information criteria (AIC) and the Bayesian information criteria (BIC); the model with the lowest values was considered to have the best fit. Genotype differences and other statistical analyses, including chi-square tests, independent t tests, and logistic regression analysis, were performed using SPSS software version 20.0 (SPSS, Chicago, IL, USA). Data are expressed as means ± SD. Categorical data are expressed as frequencies or percentages. Associations with a two-tailed p value < 0.05 were considered statistically significant.

## Results

### The genotype frequency of two SNPs differed significantly between the PCOS group and the control group

Prior to the case-control study, we confirmed that all of the genotyped SNPs agreed with Hardy-Weinberg equilibrium, thus ensuring that our sampling is representative of a truly large population (MAF: minor allele frequency > 0.01, Hardy–Weinberg equilibrium p > 0.001 (the independent sample t test), call rate > 95%,). The distribution of the genotypes and allelic frequencies of the 13 SNPs were analyzed with chi-square tests, and we investigated the 13 SNPs of the samples from PCOS and control groups in five genetic models: the log-additive model, the dominant model, the recessive model, the co-dominant model, and the overdominant model.

Significant differences were detected for two SNPs: rs1360780 and rs3800373 (Table 3). The rs1360780 genotype frequencies for CC, CT, and TT were 54.2% (425/775), 41.3% (324/775), and 4.5% (35/775) in the PCOS patients, versus 55.2% (432/783), 37.4% (293/783), and 7.4% (58/783) in the healthy controls. Under the co-dominant genetic model, the frequencies of the three genotypes differed significantly between the PCOS patients and healthy controls (p = 0.025, AIC = 2717, BIC = 2187). Under the recessive genetic model, there were more significant carriers of the C allele in the PCOS group (95.5% (749/775) than in the control group (92.6% (725/783) (p = 0.013, AIC = 2170.2, BIC = 2180.9).

**Table 3:** Genetic models analysis of associations between the genotypes of *FKBP5* rs1360780 and rs3800373 with PCOS

SNPs	Model	Genotype	PCOS	Control	OR (95% CI)	p-value	AIC	BIC
rs1360780	Co-dominant	C/C	425 (54.2%)	432 (55.2%)	1	0.03	2717	2187
		C/T	324 (41.3%)	293 (37.4%)	0.89 (0.72–1.09)			
		T/T	35 (4.5%)	58 (7.4%)	**1.63 (1.05–2.53)**			
	Dominant	C/C	425 (54.2%)	432 (55.2%)	1	0.70	2176.2	2186.9
		C/T-T/T	359 (45.8%)	351 (44.8%)	0.96 (0.79–1.17)			
	Recessive	C/C–C/T	749 (95.5%)	725 (92.6%)	1	0.01	2170.2	2180.9
		T/T	35 (4.5%)	58 (7.4%)	**1.71 (1.11–2.64)**			
	Overdominant	C/C-T/T	460 (58.7%)	490 (62.6%)	1	0.11	2173.8	2184.5
		C/T	324 (41.3%)	293 (37.4%)	0.85 (0.69–1.04)			
	Log-additive	–	–	–	1.06 (0.90–1.24)	0.52	2175.9	2186.6
rs3800373	Co-dominant	A/A	430 (55%)	432 (56%)	1	0.03	2151.8	2167.8
		C/A	315 (40.3%)	280 (36.3%)	0.88 (0.70–1.10)			
		C/C	37 (4.7%)	59 (7.7%)	**1.59 (1.03–2.45)**			
	Dominant	A/A	430 (55%)	432 (56%)	1	0.68	2156.7	2167.8
		C/A-C/C	352 (45%)	339 (44%)	0.96 (0.78–1.17)			
	Recessive	A/A-C/A	745 (95.3%)	712 (92.3%)	1	0.02	2151.1	2161.8
		C/C	37 (4.7%)	59 (7.7%)	**1.67 (1.09–2.55)**			
	Overdominant	A/A-C/C	467 (59.7%)	491 (63.7%)	1	0.11	2154.3	2164.9
		C/A	315 (40.3%)	280 (36.3%)	0.85 (0.69–1.04)			
	Log-additive	–	–	–	1.05 (0.89–1.24)	0.55	2156.5	2167.2

OR and 95% CI in bold indicates statistical significance

*OR* odds ratio, *AIC* Akaike Information Criterion, *BIC* Bayesian information criterion

The association between each SNP and the susceptibility to HA was evaluated by calculating the odds ratio (OR) with their 95% confidence interval (95% CI) with a logistic regression analysis under five genetic models (the co-dominant model, the dominant model, the recessive model, the overdominant model and the log-additive model)

For rs3800373, the frequencies of AA, CA, and CC were 55% (430/775), 40.3% (315/775), and 4.7% (37/775) in the PCOS patients, versus 56% (432/783), 36.3% (280/783), and 4.7% (59/783) in the healthy controls, with significant differences under the co-dominant genetic model (p = 0.029, AIC = 2151.8, BIC = 2167.8). In the recessive genetic model, the frequencies of genotype AA and CA (A allele carrier) in PCOS patients and healthy control also showed a significant difference (p = 0.016, AIC = 2151.1, BIC = 2161.8). Regarding allele frequencies, no significant differences were found for any of the 13SNPs between the PCOS group and the control group (p > 0.05) (Additional file1: Table S1). Notably, none of the heterozygous conditions for the two SNPs (rs1360780 and rs3800373) showed any association between PCOS and control group.

### The genotype frequency of five snps differed significantly between the HA subtype and the NHA subtype

Recall that the PCOS patients can be divided into two groups: the hyperandrogenism (HA) and non-hyperandrogenism (NHA) subtypes. Clinical or biochemical hyperandrogenism was defined on the basis of hirsutism (modified Ferriman-Gallwey score ≥ 6) or elevated circulating total testosterone ≥ 60 ng/dl. Our study analyzed 588 HA and 187 NHA PCOS patients. The allele frequencies and genotype frequencies of the 13 SNPs were tested, and the genotype frequency of the 13 SNPs was analyzed with five genetic models (Table 4).

**Table 4:** Genotype frequencies and genetic model analysis of *FKBP5* SNPs in the HA PCOS patients group and the NHA PCOS patients group

SNPs	Model	Genotype	HA	NHA	OR (95% CI)	p-value	AIC	BIC
rs1360780	Codominant	C/C	328 (55.8%)	91 (48.7%)	1	0.09	857.7	871.6
		C/T	238 (40.5%)	83 (44.4%)	1.26 (0.89–1.77)			
		T/T	22 (3.7%)	13 (7%)	**2.13 (1.03–4.39)**			
	Dominant	C/C	328 (55.8%)	91 (48.7%)	1	0.09	857.6	866.9
		C/T-T/T	260 (44.2%)	96 (51.3%)	1.33 (0.96–1.85)			
	Recessive	C/C–C/T	566 (96.3%)	174 (93%)	1	0.08	857.4	866.7
		T/T	22 (3.7%)	13 (7%)	1.92 (0.95–3.90)			
	Overdominant	C/C-T/T	350 (59.5%)	104 (55.6%)	1	0.35	859.6	868.9
		C/T	238 (40.5%)	83 (44.4%)	1.17 (0.84–1.64)			
	Log-additive	–	–	–	**1.35 (1.02–1.77)**	0.04	856.1	865.4
rs9470080	Codominant	C/C	268 (45.7%)	71 (38%)	1	0.09	856.5	870.5
		T/C	270 (46.1%)	93 (49.7%)	1.30 (0.91–1.85)			
		T/T	48 (8.2%)	23 (12.3%)	**1.81 (1.03–3.17)**			
	Dominant	C/C	268 (45.7%)	71 (38%)	1	0.06	855.9	865.2
		T/C-T/T	318 (54.3%)	116 (62%)	1.38 (0.98–1.93)			
	Recessive	C/C-T/C	538 (91.8%)	164 (87.7%)	1	0.10	856.7	866
		T/T	48 (8.2%)	23 (12.3%)	1.57 (0.93–2.66)			
	Overdominant	C/C-T/T	316 (53.9%)	94 (50.3%)	1	0.38	858.6	867.9
		T/C	270 (46.1%)	93 (49.7%)	1.16 (0.83–1.61)			
	Log-additive	–	–	–	**1.33 (1.03–1.71)**	0.03	854.5	863.8
rs9296158	Codominant	G/G	276 (47%)	72 (38.5%)	1	0.09	857.2	871.1
		G/A	266 (45.3%)	95 (50.8%)	1.37 (0.97–1.94)			
		A/A	45 (7.7%)	20 (10.7%)	1.70 (0.95–3.06)			
	Dominant	G/G	276 (47%)	72 (38.5%)	1	0.04	855.7	865
		G/A-A/A	311 (53%)	115 (61.5%)	**1.42 (1.01–1.98)**			
	Recessive	G/G-G/A	542 (92.3%)	167 (89.3%)	1	0.20	858.3	867.6
		A/A	45 (7.7%)	20 (10.7%)	1.44 (0.83–2.51)			
	Overdominant	G/G-A/A	321 (54.7%)	92 (49.2%)	1	0.19	858.2	867.5
		G/A	266 (45.3%)	95 (50.8%)	1.25 (0.90–1.73)			
	Log-additive	–	–	–	**1.33 (1.03–1.72)**	0.03	855.2	864.6
rs1043805	Codominant	A/A	398 (67.9%)	116 (62%)	1	0.03	854.5	868.5
		T/A	174 (29.7%)	59 (31.6%)	1.16 (0.81–1.67)			
		T/T	14 (2.4%)	12 (6.4%)	**2.94 (1.32–6.53)**			
	Dominant	A/A	398 (67.9%)	116 (62%)	1	0.14	857.2	866.5
		T/A-T/T	188 (32.1%)	71 (38%)	1.30 (0.92–1.83)			
	Recessive	A/A-T/A	572 (97.6%)	175 (93.6%)	1	0.01	853.2	862.5
		T/T	14 (2.4%)	12 (6.4%)	**2.80 (1.27–6.17)**			
	Overdominant	A/A-T/T	412 (70.3%)	128 (68.5%)	1	0.63	859.1	868.4
		T/A	174 (29.7%)	59 (31.6%)	1.09 (0.76–1.56)			
	Log-additive	–	–	–	**1.37 (1.03–1.83)**	0.03	854.9	864.2
rs7757037	Codominant	A/A	225 (38.3%)	56 (29.9%)	1	0.07	856.7	870.6
		G/A	285 (48.5%)	98 (52.4%)	1.38 (0.95–2.00)			
		G/G	77 (13.1%)	33 (17.6%)	**1.72 (1.04–2.84)**			
	Dominant	A/A	225 (38.3%)	56 (29.9%)	1	0.04	855.5	864.8
		G/A-G/G	362 (61.7%)	131 (70%)	**1.45 (1.02–2.07)**			
	Recessive	A/A-G/A	510 (86.9%)	154 (82.3%)	1	0.13	857.6	866.9
		G/G	77 (13.1%)	33 (17.6%)	1.42 (0.91–2.22)			
	Overdominant	A/A-G/G	302 (51.5%)	89 (47.6%)	1	0.36	859.1	868.4
		G/A	285 (48.5%)	98 (52.4%)	1.17 (0.84–1.62)			
	Log-additive	–	–	–	**1.32 (1.04–1.69)**	0.02	854.8	864.1

OR and 95% CI in bold indicates statistical significance

*HA* hyperandrogenemia, *NHA* non-hyperandrogenemia, *OR* odds ratio, *AIC* Akaike Information Criterion. *BIC* Bayesian information criterion

The association between each SNP and the susceptibility to HA was evaluated by calculating the odds ratio (OR) with their 95% confidence interval (95% CI) with a logistic regression analysis under five genetic models (the co-dominant model, the dominant model, the recessive model, the overdominant model and the log-additive model)

Using the log-additive model, significant differences were found for all five SNPs: rs1360780 (OR is 1.35(1.02–1.77), p = 0.036), rs9470080(OR is 1.33(1.03–1.71), p = 0.028), rs9296158(OR is 1.33(1.03–1.72), p = 0.031), rs1043805(OR is 1.37(1.03–1.83), p = 0.034) and rs7757037(OR is 1.32(1.04–1.69), p = 0.023). Under the codominant genetic model, the proportions of each genotype are shown on the Table 4, the PCOS cases with following homozygous genotypes displayed a significantly lower level of testosterone: the TT genotype of rs1360780, the TT genotype of rs9470080, the TT genotype of rs1043805 and the GG genotype of rs7705037 (ORs are 2.13 (1.03–4.39), 1.81 (1.03–3.17), 2.94 (1.32–6.53) and 1.72 (1.04–2.84), and p values are 0.09, 0.09, 0.03 and 0.07 respectively).

Under a dominant genetic model, analysis of rs9296158 showed that the frequencies of GG and GA + AA were 47% (276/587) and 53% (311/587), and 38.5% (72/187) and 61.5% (115/187) in the HA PCOS patients and in the NHA PCOS patients, respectively. Notably, the genotypes carrying A alleles (G/A-A/A) were more strongly associated with NHA (OR value of 1.42 (1.01–1.98) and p-value of 0.041 under the dominant model). For rs7757037, the frequencies of AA and GA + GG were 38.3% (225/587) and 61.7% (362/587) in the HA PCOS patients, and 29.9% (56/187), and 70% (131/187) in the NHA PCOS patients. The genotypes carrying G alleles (G/A-G/G) were more strongly associated with NHA under the dominant genetic model (OR is 1.45 (1.02–2.07), p = 0.036, AIC = 855.5, BIC = 864.8). Under a recessive genetic model, analysis of rs1043805 showed that the frequencies of AA + TA and TT were 97.6% (572/586) and 14%(14/586), and 93.6%(175/187) and 6.4% (12/187) in the HA PCOS patients and in the NHA PCOS patients, respectively, OR is 2.80 (1.27–6.17) and p value is 0.01. In addition, the genotype frequencies and genetic model analysis of each SNP between the insulin resistance(IR) group and the control group were conducted, while no significant differences were found for any of the 13SNPs between the two group (P > 0.05) (Additional file 2: Table S2).

## Discussion

In this study, we evaluated potential genetic influences of 13 SNPs of androgen-responsive gene *FKBP5* in Han Chinese women with PCOS, with a particular focus on the hyperandrogenism PCOS subtype. The 13 SNPs were selected based on previous reports and database information. The frequencies of the TT genotype of rs1360780 and the CC genotype of rs3800373 were significantly lower in the PCOS patients than in the healthy controls. And we detected five *FKBP5* SNPs that showed significant differences in genotype frequency analysis: the PCOS cases showing an elevated frequency for the TT genotype of rs1360780, TT genotype of rs9470080, TT genotype of rs1043805, GG genotype of rs7757037 and GA and AA of rs9296158 genotypes displayed lower level of testosterone. Our study showed that the *FKBP5* variations in combination with PCOS, with two SNP genotypes associated with PCOS generally and five for the hyperandrogenism subtype specifically, while this kind of marginal association of the *FKBP5* rs1360780 and rs3800373 with PCOS patients and *FKBP5* rs1360780, rs9470080, rs9296158, rs1043805, rs7757037 with hyperandrogenism subtype need to be viewed with caution before further validation and subsequent experimentation are conducted.

In recent years, a large number of studies have shown that genetic factors play a role in the etiology of PCOS [25, 32, 33], the contribution of heritability of this disorder has been explained based on the approaches used in the twin and linkage studies [20]. In a discovery cohort, *FKBP4* (a kind of androgen receptor gene) SNPs rs2968909 and rs4409904 were associated with lower odds of PCOS [34]. Among the multiple SNPs associated with PCOS [35, 36], an example is a SNP at exon 17 of insulin receptor gene (*INSR*), for which the CC genotype showed a higher frequency in PCOS women than in controls [37]. Additionally, PCOS patients show an elevated frequency for the G allele of a SNP in *sorbin and SH3-domain-containing-1* (*SORBS1*), which encodes a protein known to function in both insulin resistance and glucose uptake [38]. Additionally, a strong association between follistatin and the *Cytochrome P450 Family 11 Subfamily A Member 1* (*CYP11A1*) gene in affected siblings with hyperandrogenism and PCOS related traits was predicted by studying 37 candidate genes [20].

Whereas these two previous studies linked PCOS to SNPs in genes related to glucose metabolism and insulin resistance, we focused on *FKBP5*, which is known to function as an androgen receptor. While hyperandrogenism is understood as a common clinical feature in PCOS patients, the specific contributions of androgens and related events in PCOS and PCOS-related complications are not yet clear [11]. Unlike most studies—which have compared the PCOS group with a suitable healthy control group, our present study additionally compared the hyperandrogenism and non-hyperandrogenism subtypes of PCOS patients. We detected differences between the hyperandrogenism and non-hyperandrogenism PCOS subtype groups for five SNP alleles of the *FKBP5* gene. The frequencies of TT genotype of rs1360780, the TT genotype of rs9470080, the TT genotype of rs1043805 or the GG genotype of rs7705037 were significantly higher in the NHA PCOS group than that in the HA PCOS group. Regarding genetic model analysis, the AA genotype frequency of the HA group is higher than that in the HA group in the dominant model for rs7757037, and for rs9296158, the GG genotype showed the same trend in the dominant model. While for rs9470080, the frequency of the TT genotype in NHA is higher than that in the HA group. Previous reports have proposed that the effects of androgens on cell activities in PCOS may relate to apoptosis, autophagy, mitochondrial dysfunction, and/or endoplasmic reticulum stress in granule cells and oocytes [11]. Excessive androgens could aggravate the development of hypertension and atherosclerosis in patients with PCOS [39]. Particularly, previous studies examined the association of PCOS with genes involved in androgen biosynthesis and action, which supports the hypothesis that inherited abnormalities in genes involved in androgen signaling may contribute to PCOS [34].

*FKBP5* is associated with endocrine disorders, which are highly consistent with the symptoms of PCOS. *FKBP5* is involved in the regulation of glucose homeostasis through its regulation of AKT2 signaling [23]. Reports have suggested that SNPs within the *FKBP5* gene may be linked to the susceptibility to develop insulin resistance and dyslipidemia [40]. *FKBP5* functions in responses to steroids, and *FKBP5* is strongly transcriptionally activated by androgens, glucocorticoids, and progestins [41]; Several polymorphisms in the *FKBP5* gene have been associated with differences in glucocorticoid sensitivity; notably many of the clinically linked SNPs localize close to (or overlap with) regions that encompass steroid-regulated enhancers [42]. The polymorphisms may contribute to the steroid up-regulation of *FKBP5* and could thereby influence the complex regulatory loops of steroid signaling [42]. Also, a study by Ortiz and colleagues reported an association between *FKBP5* intronic methylation and the risk of cardiovascular disease in T2D patients [43].

Possible functional significance of *FKBP5* polymorphisms includes association of rs1360780 with increased protein levels [44] and differences in chromatin conformation [45] due to its location in the region adjacent to the hormone response element (HRE) binding sequence. And a study examining immune cells reported that higher *FKBP5* levels promotes inflammation by activating the master immune regulator NF-κB [46]. We paid attention to *FKBP5* because of its function as an androgen receptor gene, our study showed *FKBP5* gene polymorphisms are associated with PCOS generally (rs1360780, rs3800373) and with the hyperandrogenism subtype specifically (rs1360780, rs9470080, rs9296158, rs1043805, rs7757037), among which rs3800373 and rs1043805 belong to 3’ UTR variant of *FKBP5* gene. There may be *FKBP5* variants exhibiting yet to be discovered interaction effects with PCOS. Since this is the first study investigating the role of *FKBP5* single nuclear polymorphism in PCOS it is not possible to compare our results with the data in literature. While in a discovery cohort, *FKBP4* (a kind of androgen receptor gene) SNPs rs2968909 and rs4409904 were associated with lower odds of PCOS [34].

Another study which focused on the relationship between *FKBP5* SNPs and PTSD reported that the rs9470080 TT genotype carriers had a higher risk of developing high co-occurring PTSD and depression symptoms than the C allele carriers [30]. A previous study showed that participants exposed to childhood abuse and carrying the TT genotype of the *FKBP5* SNP rs1360780 had an increased susceptibility to stress-related disorders [47]. In our study, inversely, the frequency of the TT genotype of *FKBP5* SNP rs9470080 and the TT genotype of rs1360780 was significantly higher in PCOS NHA group than PCOS HA group, and the TT of rs1043805, the GG of rs7757037 showed the same tendency. We mentioned above the SNPs of *FKBP4* rs2968909 and rs4409904 were associated with lower odds of PCOS [34]. As noted above, there are two homozygous genotypes between PCOS and control group (the TT of rs1360780 and the CC of rs3800373) and four homozygous genotypes between NHA and HA group (the TT of rs1360780, the TT of rs9470080, the TT of rs1043805, the GG of rs7757037) showing associations. For these SNPs, only the homozygous mutant might be associated with PCOS and hyperandrogenism risk.

In another study [17], more comprehensive clinical data were incorporated into an association analysis, including prolactin, blood lipid (CHOL total cholesterol, low-density lipoprotein cholesterol LDL-C, HDL-C high-density lipoprotein cholesterol, triglycerides TG, etc.), OGTT (oral glucose tolerance test), FPG (fasting plasma glucose), HOMA-IR (homeostasis model assessment-insulin resistance) and other clinical indices. Note that we were particularly focused on the hyperandrogenism symptom in our case–control study.

There are some limitations to this case–control study that bear mention. First, the participants were all Han Chinese women, so there was very little variation in genetic background. Genetic factors that influence the pathogenesis of PCOS are known to differ by ethnic group [33, 48], so the associations we detected between *FKBP5* and PCOS need to be confirmed in a larger sample size that includes multiple ethnic groups. Second, the functional significance of the SNPs remains unknown and molecular mechanisms regarding *FKBP5* in the pathophysiology of PCOS should be examined in future studies.

Our study covered a relatively large cohort of Chinese women with PCOS and represents the first study providing data about the *FKBP5* gene and PCOS. On the basis of the available evidence, we conclude that the SNPs, rs1360780, rs3800373, rs9470080, rs9296158, rs1043805, and rs7757037 in the *FKBP5* gene are strongly associated with PCOS among Han Chinese women. These SNPs we detected should be useful in future investigations of HA in PCOS, and especially for supporting clinical diagnosis of PCOS as informed by genetic testing. The major limitation of our study was the small number of the studied sample. Further studies in humans and potentially some functional studies will be required to validate the predictive value of these SNPs as potential biomarkers and to elucidate the effects of *FKBP5* on PCOS development and clinical outcomes.

## Conclusions

Our study showed that the *FKBP5* gene is a potential association gene for PCOS, with some SNP genotypes associated with PCOS generally and with the hyperandrogenism subtype specifically. On the basis of the available evidence, we conclude that the SNPs, rs1360780, rs3800373, rs9470080, rs9296158, rs1043805, and rs7757037 in the *FKBP5* gene are strongly associated with PCOS among Han Chinese women. Assuming the further validation including functional significance and molecular mechanisms of the SNPs and other evidence appears, we indicate that the SNPs we detected will be useful in future investigations of HA in PCOS, especially for supporting clinical diagnosis of PCOS as informed by genetic testing.

## Supplementary Information

**Additional file1**. **Table S1**: Allele frequencies of *FKBP5* SNPs in the PCOS patients and in healthy controls.

**Additional file2.** **Table S2**: Genotype frequencies and genetic model analysis of *FKBP5* SNPs in the IR group and the control group.

## Data Availability

All data supporting the findings of this study are available within the manuscript except for the raw sequence data. Any data providing genotype information is considered to be personal property by Chinese law, hence the submission to public achieves is prohibited. The raw sequence data can be acquired upon reasonable request from the authors (yrzhao@sdu.edu.cn), if approval could be granted from the Ethics Committee of Reproductive Medicine of Shandong University.

## Abbreviations

PCOS: Polycystic ovary syndrome
*FKBP5*: FK-506 binding protein 5
SNP: Single-nucleotide polymorphisms
HA: Hyperandrogenism
NHA: Non-hyperandrogenism
BMI: Body mass index
AR: Androgen receptor
T2D: Type 2 diabetes
FSH: Follicle-stimulating hormone
LH: Luteinizing hormone
T: Testosterone
MAF: Minor allele frequency
AIC: Akaike information criteria
BIC: Bayesian information criteria
