# Supplementary material for: Association of SNPs in the FK-506 binding protein (FKBP5) gene among Han Chinese women with polycystic ovary syndrome
Source: BMC Med Genomics. 2022 Jul 4;15:149. doi: 10.1186/s12920-022-01301-0 (PMC9254403; doi:10.1186/s12920-022-01301-0)
Supplement: Supplementary file 1 — Additional file1. Table S1: Allele frequencies of FKBP5 SNPs in the PCOS patients and in healthy controls. [file 12920_2022_1301_MOESM1_ESM.docx]

**Supplementary Table 1 Allele frequencies of *FKBP5* SNPs in the PCOS patients and in healthy controls**

| SNP | Minor allele | Minor allele frequency | | OR (95%CI) | P |
| --- | --- | --- | --- | --- | --- |
|  |  | PCOS | CON |  |  |
| rs1360780 | T | 0.25 | 0.26 | 0.96（0.80**-**1.14） | 0.62 |
| rs3800373 | C | 0.25 | 0.25 | 0.95（0.80**-**1.13） | 0.54 |
| rs9296158 | A | 0.32 | 0.33 | 0.94（0.80**-**1.11） | 0.45 |
| rs9470080 | T | 0.32 | 0.34 | 0.93（0.79**-**1.10） | 0.41 |
| rs2817035 | A | 0.22 | 0.23 | 0.92（0.76**-**1.11） | 0.36 |
| rs3798346 | G | 0.05 | 0.05 | 0.95（0.67**-**1.35） | 0.78 |
| rs4713902 | C | 0.27 | 0.25 | 1.10（0.92**-**1.31） | 0.29 |
| rs4713916 | A | 0.22 | 0.22 | 1.00（0.83**-**1.21） | 0.97 |
| rs755658 | T | 0.07 | 0.08 | 0.87（0.65**-**1.16） | 0.33 |
| rs7757037 | G | 0.37 | 0.39 | 0.94（0.80**-**1.10） | 0.46 |
| rs9394309 | G | 0.21 | 0.21 | 0.99（0.82**-**1.19） | 0.90 |
| rs1043805 | T | 0.18 | 0.18 | 0.99（0.81**-**1.20） | 0.89 |
| rs1475774 | A | 0.08 | 0.07 | 1.02（0.77**-**1.37） | 0.88 |

hyperandrogenemia, NHA, non-hyperandrogenemia.
