# Supplementary material for: Association of SNPs in the FK-506 binding protein (FKBP5) gene among Han Chinese women with polycystic ovary syndrome
Source: BMC Med Genomics. 2022 Jul 4;15:149. doi: 10.1186/s12920-022-01301-0 (PMC9254403; doi:10.1186/s12920-022-01301-0)
Supplement: Supplementary file 2 — Additional file2. Table S2: Genotype frequencies and genetic model analysis of FKBP5 SNPs in the IR group and the control group. [file 12920_2022_1301_MOESM2_ESM.docx]

**Supplementary Table 2 Genotype Frequencies and Genetic Model Analysis of *FKBP5* SNPs in the IR group and the Control group**

| SNPs | Model | Genotype | IR | Con | OR (95% CI) | P-value | AIC | BIC |
| --- | --- | --- | --- | --- | --- | --- | --- | --- |
| rs1360780 | Codominant | C/C | 313 (53.6%) | 432 (55.2%) | 1 | 0.094 | 1867.3 | 1882.9 |
|  |  | C/T | 242 (41.4%) | 293 (37.4%) | 0.88 (0.70-1.10) |  |  |  |
|  |  | T/T | 29 (5%) | 58 (7.4%) | 1.45 (0.91-2.32) |  |  |  |
|  | Dominant | C/C | 313 (53.6%) | 432 (55.2%) | 1 | 0.56 | 1869.7 | 1880.1 |
|  |  | C/T-T/T | 271 (46.4%) | 351 (44.8%) | 0.94 (0.76-1.16) |  |  |  |
|  | Recessive | C/C-C/T | 555 (95%) | 725 (92.6%) | 1 | 0.064 | 1866.6 | 1877 |
|  |  | T/T | 29 (5%) | 58 (7.4%) | 1.53 (0.97-2.42) |  |  |  |
|  | Overdominant | C/C-T/T | 342 (58.6%) | 490 (62.6%) | 1 | 0.13 | 1867.7 | 1878.2 |
|  |  | C/T | 242 (41.4%) | 293 (37.4%) | 0.85 (0.68-1.05) |  |  |  |
|  | Log-additive | --- | --- | --- | 1.02 (0.86-1.22) | 0.8 | 1869.9 | 1880.4 |
| rs3800373 | Codominant | A/A | 315 (54%) | 432 (56%) | 1 | 0.098 | 1852.2 | 1867.8 |
|  |  | C/A | 237 (40.6%) | 280 (36.3%) | 0.86 (0.69-1.08) |  |  |  |
|  |  | C/C | 31 (5.3%) | 59 (7.7%) | 1.39 (0.88-2.19) |  |  |  |
|  | Dominant | A/A | 315 (54%) | 432 (56%) | 1 | 0.46 | 1854.3 | 1864.7 |
|  |  | C/A-C/C | 268 (46%) | 339 (44%) | 0.92 (0.74-1.14) |  |  |  |
|  | Recessive | A/A-C/A | 552 (94.7%) | 712 (92.3%) | 1 | 0.084 | 1851.9 | 1862.3 |
|  |  | C/C | 31 (5.3%) | 59 (7.7%) | 1.48 (0.94-2.31) |  |  |  |
|  | Overdominant | A/A-C/C | 346 (59.4%) | 491 (63.7%) | 1 | 0.1 | 1852.2 | 1862.6 |
|  |  | C/A | 237 (40.6%) | 280 (36.3%) | 0.83 (0.67-1.04) |  |  |  |
|  | Log-additive | --- | --- | --- | 1.01 (0.85-1.20) | 0.92 | 1854.8 | 1865.3 |
| rs9296158 | Codominant | G/G | 260 (44.5%) | 352 (45%) | 1 | 0.49 | 1870.6 | 1886.2 |
|  |  | G/A | 271 (46.4%) | 346 (44.2%) | 0.94 (0.75-1.18) |  |  |  |
|  |  | A/A | 53 (9.1%) | 85 (10.9%) | 1.18 (0.81-1.73) |  |  |  |
|  | Dominant | G/G | 260 (44.5%) | 352 (45%) | 1 | 0.87 | 1870 | 1880.4 |
|  |  | G/A-A/A | 324 (55.5%) | 431 (55%) | 0.98 (0.79-1.22) |  |  |  |
|  | Recessive | G/G-G/A | 531 (90.9%) | 698 (89.1%) | 1 | 0.28 | 1868.8 | 1879.3 |
|  |  | A/A | 53 (9.1%) | 85 (10.9%) | 1.22 (0.85-1.75) |  |  |  |
|  | Overdominant | G/G-A/A | 313 (53.6%) | 437 (55.8%) | 1 | 0.42 | 1869.3 | 1879.8 |
|  |  | G/A | 271 (46.4%) | 346 (44.2%) | 0.91 (0.74-1.13) |  |  |  |
|  | Log-additive | --- | --- | --- | 1.03 (0.88-1.22) | 0.71 | 1869.9 | 1880.3 |
| rs9470080 | Codominant | C/C | 255 (43.7%) | 339 (44%) | 1 | 0.35 | 1853.6 | 1869.3 |
|  |  | T/C | 271 (46.5%) | 338 (43.9%) | 0.94 (0.75-1.18) |  |  |  |
|  |  | T/T | 57 (9.8%) | 93 (12.1%) | 1.23 (0.85-1.77) |  |  |  |
|  | Dominant | C/C | 255 (43.7%) | 339 (44%) | 1 | 0.92 | 1853.7 | 1864.1 |
|  |  | T/C-T/T | 328 (56.3%) | 431 (56%) | 0.99 (0.80-1.23) |  |  |  |
|  | Recessive | C/C-T/C | 526 (90.2%) | 677 (87.9%) | 1 | 0.18 | 1851.9 | 1862.3 |
|  |  | T/T | 57 (9.8%) | 93 (12.1%) | 1.27 (0.89-1.80) |  |  |  |
|  | Overdominant | C/C-T/T | 312 (53.5%) | 432 (56.1%) | 1 | 0.34 | 1852.8 | 1863.3 |
|  |  | T/C | 271 (46.5%) | 338 (43.9%) | 0.90 (0.73-1.12) |  |  |  |
|  | Log-additive | --- | --- | --- | 1.05 (0.89-1.23) | 0.58 | 1853.4 | 1863.8 |
| rs2817035 | Codominant | G/G | 340 (58.2%) | 455 (58.1%) | 1 | 0.67 | 1871.2 | 1886.9 |
|  |  | G/A | 220 (37.7%) | 288 (36.8%) | 0.98 (0.78-1.22) |  |  |  |
|  |  | A/A | 24 (4.1%) | 40 (5.1%) | 1.25 (0.74-2.11) |  |  |  |
|  | Dominant | G/G | 340 (58.2%) | 455 (58.1%) | 1 | 0.97 | 1870 | 1880.4 |
|  |  | G/A-A/A | 244 (41.8%) | 328 (41.9%) | 1.00 (0.81-1.25) |  |  |  |
|  | Recessive | G/G-G/A | 560 (95.9%) | 743 (94.9%) | 1 | 0.38 | 1869.2 | 1879.7 |
|  |  | A/A | 24 (4.1%) | 40 (5.1%) | 1.26 (0.75-2.11) |  |  |  |
|  | Overdominant | G/G-A/A | 364 (62.3%) | 495 (63.2%) | 1 | 0.74 | 1869.9 | 1880.3 |
|  |  | G/A | 220 (37.7%) | 288 (36.8%) | 0.96 (0.77-1.20) |  |  |  |
|  | Log-additive | --- | --- | --- | 1.03 (0.86-1.24) | 0.73 | 1869.9 | 1880.3 |
| rs3798346 | Codominant | A/A | 515 (88.2%) | 682 (89.7%) | 1 | 0.56 | 1844.9 | 1860.5 |
|  |  | G/A | 66 (11.3%) | 76 (10%) | 0.87 (0.61-1.23) |  |  |  |
|  |  | G/G | 3 (0.5%) | 2 (0.3%) | 0.50 (0.08-3.02) |  |  |  |
|  | Dominant | A/A | 515 (88.2%) | 682 (89.7%) | 1 | 0.37 | 1843.3 | 1853.7 |
|  |  | G/A-G/G | 69 (11.8%) | 78 (10.3%) | 0.85 (0.61-1.20) |  |  |  |
|  | Recessive | A/A-G/A | 581 (99.5%) | 758 (99.7%) | 1 | 0.46 | 1843.5 | 1853.9 |
|  |  | G/G | 3 (0.5%) | 2 (0.3%) | 0.51 (0.09-3.07) |  |  |  |
|  | Overdominant | A/A-G/G | 518 (88.7%) | 684 (90%) | 1 | 0.44 | 1843.5 | 1853.9 |
|  |  | G/A | 66 (11.3%) | 76 (10%) | 0.87 (0.62-1.24) |  |  |  |
|  | Log-additive | --- | --- | --- | 0.85 (0.61-1.17) | 0.32 | 1843.1 | 1853.5 |
| rs4713902 | Codominant | T/T | 326 (55.8%) | 441 (56.4%) | 1 | 0.95 | 1870.8 | 1886.4 |
|  |  | C/T | 222 (38%) | 291 (37.2%) | 0.97 (0.77-1.21) |  |  |  |
|  |  | C/C | 36 (6.2%) | 50 (6.4%) | 1.03 (0.65-1.61) |  |  |  |
|  | Dominant | T/T | 326 (55.8%) | 441 (56.4%) | 1 | 0.83 | 1868.8 | 1879.3 |
|  |  | C/T-C/C | 258 (44.2%) | 341 (43.6%) | 0.98 (0.79-1.21) |  |  |  |
|  | Recessive | T/T-C/T | 548 (93.8%) | 732 (93.6%) | 1 | 0.86 | 1868.8 | 1879.3 |
|  |  | C/C | 36 (6.2%) | 50 (6.4%) | 1.04 (0.67-1.62) |  |  |  |
|  | Overdominant | T/T-C/C | 362 (62%) | 491 (62.8%) | 1 | 0.76 | 1868.8 | 1879.2 |
|  |  | C/T | 222 (38%) | 291 (37.2%) | 0.97 (0.77-1.21) |  |  |  |
|  | Log-additive | --- | --- | --- | 0.99 (0.83-1.18) | 0.92 | 1868.9 | 1879.3 |
| rs4713916 | Codominant | G/G | 345 (59.1%) | 479 (61.2%) | 1 | 0.58 | 1870.9 | 1886.6 |
|  |  | G/A | 213 (36.5%) | 265 (33.8%) | 0.90 (0.71-1.12) |  |  |  |
|  |  | A/A | 26 (4.5%) | 39 (5%) | 1.08 (0.65-1.81) |  |  |  |
|  | Dominant | G/G | 345 (59.1%) | 479 (61.2%) | 1 | 0.43 | 1869.4 | 1879.8 |
|  |  | G/A-A/A | 239 (40.9%) | 304 (38.8%) | 0.92 (0.74-1.14) |  |  |  |
|  | Recessive | G/G-G/A | 558 (95.5%) | 744 (95%) | 1 | 0.65 | 1869.8 | 1880.2 |
|  |  | A/A | 26 (4.5%) | 39 (5%) | 1.13 (0.68-1.87) |  |  |  |
|  | Overdominant | G/G-A/A | 371 (63.5%) | 518 (66.2%) | 1 | 0.31 | 1869 | 1879.4 |
|  |  | G/A | 213 (36.5%) | 265 (33.8%) | 0.89 (0.71-1.12) |  |  |  |
|  | Log-additive | --- | --- | --- | 0.96 (0.80-1.15) | 0.62 | 1869.8 | 1880.2 |
| rs755658 | Codominant | C/C | 512 (87.7%) | 663 (84.7%) | 1 | 0.17 | 1868.5 | 1884.1 |
|  |  | C/T | 70 (12%) | 113 (14.4%) | 1.25 (0.91-1.72) |  |  |  |
|  |  | T/T | 2 (0.3%) | 7 (0.9%) | 2.70 (0.56-13.07) |  |  |  |
|  | Dominant | C/C | 512 (87.7%) | 663 (84.7%) | 1 | 0.11 | 1867.5 | 1877.9 |
|  |  | C/T-T/T | 72 (12.3%) | 120 (15.3%) | 1.29 (0.94-1.76) |  |  |  |
|  | Recessive | C/C-C/T | 582 (99.7%) | 776 (99.1%) | 1 | 0.2 | 1868.3 | 1878.8 |
|  |  | T/T | 2 (0.3%) | 7 (0.9%) | 2.62 (0.54-12.68) |  |  |  |
|  | Overdominant | C/C-T/T | 514 (88%) | 670 (85.6%) | 1 | 0.19 | 1868.3 | 1878.7 |
|  |  | C/T | 70 (12%) | 113 (14.4%) | 1.24 (0.90-1.70) |  |  |  |
|  | Log-additive | --- | --- | --- | 1.30 (0.97-1.75) | 0.079 | 1866.9 | 1877.4 |
| rs7757037 | Codominant | A/A | 207 (35.5%) | 294 (37.6%) | 1 | 0.59 | 1869.8 | 1885.5 |
|  |  | G/A | 286 (49%) | 361 (46.2%) | 0.89 (0.70-1.13) |  |  |  |
|  |  | G/G | 91 (15.6%) | 127 (16.2%) | 0.98 (0.71-1.36) |  |  |  |
|  | Dominant | A/A | 207 (35.5%) | 294 (37.6%) | 1 | 0.41 | 1868.2 | 1878.6 |
|  |  | G/A-G/G | 377 (64.5%) | 488 (62.4%) | 0.91 (0.73-1.14) |  |  |  |
|  | Recessive | A/A-G/A | 493 (84.4%) | 655 (83.8%) | 1 | 0.74 | 1868.8 | 1879.2 |
|  |  | G/G | 91 (15.6%) | 127 (16.2%) | 1.05 (0.78-1.41) |  |  |  |
|  | Overdominant | A/A-G/G | 298 (51%) | 421 (53.8%) | 1 | 0.3 | 1867.8 | 1878.3 |
|  |  | G/A | 286 (49%) | 361 (46.2%) | 0.89 (0.72-1.11) |  |  |  |
|  | Log-additive | --- | --- | --- | 0.97 (0.83-1.13) | 0.69 | 1868.7 | 1879.2 |
| rs9394309 | Codominant | A/A | 355 (60.8%) | 490 (62.7%) | 1 | 0.55 | 1869.7 | 1885.3 |
|  |  | A/G | 204 (34.9%) | 253 (32.4%) | 0.90 (0.71-1.13) |  |  |  |
|  |  | G/G | 25 (4.3%) | 39 (5%) | 1.13 (0.67-1.90) |  |  |  |
|  | Dominant | A/A | 355 (60.8%) | 490 (62.7%) | 1 | 0.48 | 1868.4 | 1878.8 |
|  |  | A/G-G/G | 229 (39.2%) | 292 (37.3%) | 0.92 (0.74-1.15) |  |  |  |
|  | Recessive | A/A-A/G | 559 (95.7%) | 743 (95%) | 1 | 0.54 | 1868.5 | 1878.9 |
|  |  | G/G | 25 (4.3%) | 39 (5%) | 1.17 (0.70-1.96) |  |  |  |
|  | Overdominant | A/A-G/G | 380 (65.1%) | 529 (67.7%) | 1 | 0.32 | 1867.9 | 1878.3 |
|  |  | A/G | 204 (34.9%) | 253 (32.4%) | 0.89 (0.71-1.12) |  |  |  |
|  | Log-additive | --- | --- | --- | 0.97 (0.80-1.16) | 0.71 | 1868.7 | 1879.2 |
| rs1043805 | Codominant | A/A | 377 (64.7%) | 528 (67.5%) | 1 | 0.54 | 1867.9 | 1883.6 |
|  |  | T/A | 182 (31.2%) | 223 (28.5%) | 0.87 (0.69-1.11) |  |  |  |
|  |  | T/T | 24 (4.1%) | 31 (4%) | 0.92 (0.53-1.60) |  |  |  |
|  | Dominant | A/A | 377 (64.7%) | 528 (67.5%) | 1 | 0.27 | 1866 | 1876.4 |
|  |  | T/A-T/T | 206 (35.3%) | 254 (32.5%) | 0.88 (0.70-1.10) |  |  |  |
|  | Recessive | A/A-T/A | 559 (95.9%) | 751 (96%) | 1 | 0.89 | 1867.2 | 1877.6 |
|  |  | T/T | 24 (4.1%) | 31 (4%) | 0.96 (0.56-1.66) |  |  |  |
|  | Overdominant | A/A-T/T | 401 (68.8%) | 559 (71.5%) | 1 | 0.28 | 1866 | 1876.4 |
|  |  | T/A | 182 (31.2%) | 223 (28.5%) | 0.88 (0.70-1.11) |  |  |  |
|  | Log-additive | --- | --- | --- | 0.91 (0.75-1.10) | 0.33 | 1866.2 | 1876.7 |
| rs1475774 | Codominant | G/G | 498 (85.3%) | 659 (85.9%) | 1 | 0.65 | 1853.1 | 1868.8 |
|  |  | G/A | 84 (14.4%) | 103 (13.4%) | 0.93 (0.68-1.26) |  |  |  |
|  |  | A/A | 2 (0.3%) | 5 (0.6%) | 1.89 (0.37-9.78) |  |  |  |
|  | Dominant | G/G | 498 (85.3%) | 659 (85.9%) | 1 | 0.74 | 1851.9 | 1862.3 |
|  |  | G/A-A/A | 86 (14.7%) | 108 (14.1%) | 0.95 (0.70-1.29) |  |  |  |
|  | Recessive | G/G-G/A | 582 (99.7%) | 762 (99.3%) | 1 | 0.42 | 1851.4 | 1861.8 |
|  |  | A/A | 2 (0.3%) | 5 (0.6%) | 1.91 (0.37-9.88) |  |  |  |
|  | Overdominant | G/G-A/A | 500 (85.6%) | 664 (86.6%) | 1 | 0.62 | 1851.8 | 1862.2 |
|  |  | G/A | 84 (14.4%) | 103 (13.4%) | 0.92 (0.68-1.26) |  |  |  |
|  | Log-additive | --- | --- | --- | 0.98 (0.73-1.30) | 0.87 | 1852 | 1862.4 |

IR, insulin resistance, Con, control.

The association between each SNP and the susceptibility to HA was evaluated by calculating the odds ratio (OR) with their 95 % confidence interval (95 % CI) with a logistic regression analysis under five genetic models (the co-dominant model, the dominant model, the recessive model, the overdominant model and the log-additive model).
